# Systematic analysis of cuproptosis-related genes in immunological characterization and predictive drugs in Alzheimer’s disease

**DOI:** 10.3389/fnagi.2023.1204530

**Published:** 2023-10-18

**Authors:** Bin Nie, Yefen Duan, Xuelong Xie, Lihua Qiu, Shaorui Shi, Zhili Fan, Xuxiang Zheng, Ling Jiang

**Affiliations:** ^1^Department of Clinical Laboratory, The Second People’s Hospital of Yibin·West China Yibin Hospital, Sichuan University, Yibin, China; ^2^Clinical Research and Translational Center, The Second People’s Hospital of Yibin·West China Yibin Hospital, Sichuan University, Yibin, China; ^3^Department of Clinical Laboratory, Yibin No. 4 People’s Hospital, Yibin, China; ^4^Imaging Department, The Second People’s Hospital of Yibin·West China Yibin Hospital, Sichuan University, Yibin, China; ^5^Department of Neurology, The Second People’s Hospital of Yibin·West China Yibin Hospital, Sichuan University, Yibin, China

**Keywords:** Alzheimer’s disease, cuproptosis, immune microenvironment, diagnostic model, molecular docking

## Abstract

**Objectives:**

This study aimed to make a systematic analysis of cuproptosis-related genes (CRGs) in immunological characterization and predictive drugs in Alzheimer’s disease (AD) through bioinformatics and biological experiments.

**Methods:**

The molecular clusters related to CRGs and associated immune cell infiltrations in AD were investigated. The diagnostic models were constructed for AD and different AD subtypes. Moreover, drug prediction and molecular docking were also performed. Subsequently, a molecular dynamics (MD) simulation was conducted to further verify the findings. Finally, RT-qPCR validation was performed.

**Results:**

The characterization of 12 AD-related CRGs was evaluated in AD, and a diagnostic model for AD showed a satisfying discrimination power based on five CRGs by LASSO regression analysis. The dysregulated CRGs and activated immune responses partially differed between patients with AD and healthy subjects. Furthermore, two molecular subtypes (clusters A and B) with different immune infiltration characteristics in AD were identified. Similarly, a diagnostic model for different AD subtypes was built with nine CRGs, which achieved a good performance. Molecular docking revealed the optimum conformation of CHEMBL261454 and its target gene CSNK1D, which was further validated by MD simulation. The RT-qPCR results were consistent with those of the comprehensive analysis.

**Conclusion:**

This study systematically elucidated the complex relationship between cuproptosis and AD, providing novel molecular targets for treatment and diagnosis biomarkers of AD.

## Introduction

Alzheimer’s disease (AD) is the most general neurodegenerative disease among older adults, characterized by executive and visuospatial dysfunction, deficits in short-term memory, and praxis (Oboudiyat et al., 2013). The most recent available data indicates that 42.3 million people are living with AD and this number is forecast to reach 85 million by 2060 (Alzheimer’s Association, 2022). The financial burden on families due to AD is substantial. The monetary costs linked to the disease are expected to be equally significant and to increase continuously (Deb and Sambamoorthi, 2018). Moreover, due to the clinical heterogeneity and complexity of pathological types, there is a lack of satisfactory treatment and effective prevention strategies. Given the high incidence and mortality of AD, it is of great significance for the early diagnosis, prevention, and treatment of AD to understand its etiology and pathogenesis by identifying molecular biomarkers and further determining the molecular subtypes of AD at the molecular level.

Neurodegeneration in AD is likely caused by various mechanisms. These include, but are not limited to, energy and mitochondrial dysfunction, oxidative stress, cell cycle abnormalities, and neurovascular dysfunction (Blennow et al., 2006). Extensive neuronal loss is a long-standing observation and previous research has concentrated on apoptosis, but the recent identification of cuproptosis as a new type of cell death has raised significant concerns that excessive copper accumulation and the aggregation of lipoylated proteins can lead to cellular demise through proteotoxic stress (Mangalmurti and Lukens, 2022; Tsvetkov and Coy, 2022). It is well known that copper homeostasis is largely dependent on mitochondrial regulation (Baker et al., 2017). A recent study has linked tau pathology in AD to mitochondrial dysfunction (Cheng and Bai, 2018). The overexpression of hyperphosphorylated and aggregated tau may harm the axonal transport of various organelles and mitochondrial dynamics, leading to mitochondrial dysfunction (Eckert et al., 2014). Tau becomes phosphorylated and aggregated because of mitochondrial dysfunction. However, hyperphosphorylated tau disrupts mitochondrial axonal transport, which damages nerve and synaptic function and leads to memory impairment in AD (Cheng and Bai, 2018).

Hypoxia is one of the most common pathological reactions and can be induced by ischemic injury, trauma, inflammation, tumors, and other events. Hypoxia can promote cell proliferation and invasion and regulate immune response (Jing et al., 2019). One of the main regulatory factors of cell response to hypoxia is a kind of protein (Wu et al., 2019) called hypoxia-inducible factor-1 (HIF-1). Neurons in the central nervous system are particularly sensitive to hypoxia. Even transient ischemic hypoxia can lead to severe brain damage, especially in the hippocampus, which is extremely sensitive to hypoxia. Because the hippocampus is very important for spatial learning and memory, damage in this area will lead to a significant decline in cognitive function, thus seriously reducing the quality of life of patients (Yang et al., 2017). In addition, the central nervous system (CNS) contains a variety of immune cell types, which play different roles in tissue homeostasis, immune defense, and nervous system diseases. In the process of neurodegenerative diseases, inflammation in the brain gradually increases, the immune landscape of the central nervous system changes significantly, the resident immune cells are activated, and the essence can be infiltrated by inflammatory white blood cells (Deleidi et al., 2015). White blood cells in the central nervous system may also play a role in age-related inflammation and neurodegeneration (Ritzel et al., 2016). Type 1 and type 17 T cells, two subtypes of CD4+ T cells, promote the development of AD by triggering a glial pro-inflammatory reaction (McQuillan et al., 2010). The changes in dendritic cells may be related to serious clinical symptoms (Ciaramella et al., 2016). These findings emphasize the important role of immune infiltration and hypoxia levels in AD. Therefore, it is necessary to study the immune microenvironment of AD in detail and accurately identify molecular subtypes, which is helpful to determine which patients with AD can benefit from immunotherapy.

In the present study, based on bioinformatics and biological experiments, we systematically explored the influence of cuproptosis on the occurrence and development of AD from the perspective of genes, providing novel insights into the treatment of AD.

## Methods and materials

### Data acquisition

After excluding cell line or animal-level studies and single sample studies, two datasets (GSE63060 and GSE63061) were obtained from the Gene Expression Omnibus (GEO) database.^1^ The GSE63060 dataset (GPL6947 platform), including blood samples from 104 healthy controls and 145 patients with AD, was selected as the training set for further analysis. The GSE63061 dataset (GPL10558 platform), which included blood samples from 134 healthy subjects and 139 patients with AD, was selected as the validation set.

### Cuproptosis-related genes (CRGs) collection

In total, 12 CRGs were collected based on the literature review. Among them, there were 10 genes involving the cuproptosis pathway, including 7 pro-cuproptosis genes (DLD, FDX1, LIPT1, LIAS, PDHB, DLAT, and PDHA1) and 3 anti-cuproptosis genes (CDKN2A, MTF1, and GLS). Besides, SLC31A1 and ATP7B, 2 copper transporters genes, were also selected.

### Differentially expressed genes (DEGs) and AD-related differentially CRGs analysis

The DEGs with *p*-value <0.05 between AD and healthy control groups in GSE63060 were obtained via the Limma package in R (version 3.40.6).

Protein–protein interaction (PPI) networks of 12 CRGs were reviewed from the STRING database.^2^ Spearman correlation analysis was used to evaluate the relationship between the expressions of 12 CRGs in all AD samples. The expressions of 12 CRGs between patients with AD and healthy subjects were compared by the *t-*test, and the difference standard was a *p*-value of <0.05. Least absolute shrinkage and selection operator (LASSO) regression was applied for feature selection and size reduction to select diagnostic-relevant genes with non-zero coefficients to establish a diagnostic model for AD. Receiver operating characteristic (ROC) analysis was performed with the R package “pROC (version 1.15.3)” to calculate the area under the curve (AUC) to evaluate the model accuracy.

### Evaluating the immune cell infiltration and hypoxia status in different AD subtypes

The relative abundances of 23 types of immune cells in each sample as well as the activity of specific immune responses based on the proceeded gene expression data were estimated by the single sample gene set enrichment analysis (ssGSEA) algorithm. Gene sets that marked each IME infiltrating immune cell were gained from a previous study (Charoentong et al., 2017), which were rich in human immune cell subtypes, including natural killer (NK) cells, dendritic cells, CD8^+^ T cells, macrophages, NK T (NKT) cells, and Tregs cells. Immune response genes were collected from the import database.^3^ Wilcox test was used to compare the differences in immune cell infiltration, immune response activity, and HLA-related genes between different groups. Spearman correlation analysis was performed to analyze the correlation between the cuproptosis and the relative percentage of immune cells. The differences of 23 immune cells were verified in the validation set.

The unsupervised clustering analysis was utilized based on the expression profiles of the 12 CRGs, employing the k-means algorithm with 500 iterations to classify the AD samples into different clusters. With LASSO regression analysis, diagnostic models for different AD subtypes were constructed. The Wilcox test was performed to compare infiltrating immune cell abundance score, CRG expression, immune response score, and HLA gene expression for different cuproptosis modification patterns. The same method was used to classify AD into different cuproptosis-related subtypes in the validation set, and the differences in the abundance scores of infiltrating immune cells among them were verified.

In addition, since oxygen deprivation stress is a non-hereditary risk factor for AD, and cuproptosis is weakened under oxygen-deprived conditions (James et al., 2012), three hypoxia-related gene sets (HALLMARK_HYPOXIA, WINTER_HYPOXIA_DN, and WINTER_HYPOXIA_UP) were selected for GSEA analysis to assess hypoxia among different subtypes associated with cuproptosis in AD.

### Weighted gene co-expression network analysis (WGCNA)

The co-expression modules were identified by WGCNA using the “WGCNA” R package (version 1, 70.3). To ensure the quality of the results, the top 25% of genes with the highest variance were selected for subsequent WGCNA analyses. Since the modules identified by the dynamic tree-cutting algorithm may be similar, they were merged with a height cutoff of 0.25.

### Identification of hub genes and enrichment analysis

To identify the hub genes, the module connectivity of each gene, which was relative to AD subtypes based on the WGCNA algorithm, was analyzed. Module connectivity was defined as the module membership (MM), that is, the correlation of the module eigengene and the gene expression profile. Specifically, AD subtype hub genes were defined as the most relative genes in the related module. Gene ontology (GO) and Kyoto Encyclopedia of Genes and Genomes (KEGG) enrichment analyses were performed on the selected genes using DAVID.9 to better comprehend their functions. *P*-value <0.05 was considered as significantly enriched and the results of GO and KEGG enrichment analysis were plotted by a bioinformatics online tool.

### Drug prediction and molecular docking

Based on the Drug-Gene Interaction database (DGIdb^4^), drugs related to hub genes were screened, and then molecular docking was performed between candidate drugs and target genes to reveal the relationship between them. Briefly, receptor target protein structures obtained from the RCSB PDB databases^5^ and the active ingredients of drugs and their corresponding 3D structures obtained from the PubChem were imported into AutoDock Vina. Protein hydrogenation was performed using AutoDock 4.2.6 software, and PyMOL software was used for dehydration/ligand/receptor analysis. AutoDock Vina 1.1.222 was employed to dock three receptor proteins with three small molecule ligands. The binding energy was used as a reference in screening for the most active ligand molecules and target genes. A binding energy <0 meant that the ligand and receptor could bind spontaneously, with smaller values indicating a more stable binding. A binding energy below −5.0 kJ/mol was considered to indicate better binding activity for drugs (Liu et al., 2020).

### Molecular dynamics (MD) simulation

The GROMACS software package was utilized for conducting MD simulations. The protein was simulated using the CHARMM36 force field parameters, while the topology of small molecules was built based on the CGenFF database. The small molecule-protein complex was then placed in a solvent box, filled with water molecules, and stabilized with Cl^−^ and Na^+^ ions to maintain an electrically neutral system. After equilibrating the system using an NPT ensemble (fixed pressure, temperature, and particle number), the simulation time was set to 50 ns, and the simulation went through heating, equilibration, and production stages. Finally, the simulation results were analyzed based on root mean square deviation (RMSD), root mean square fluctuation (RMSF), radius of gyration (RoG), solvent accessible surface area (SASA), and hydrogen bonds (H-bonds).

### RT-qPCR validation

Total RNA from the blood samples of patients with AD (*n* = 5) and healthy subjects (*n* = 7) was extracted with a TRIzol reagent according to the manufacturer’s instructions. The patients with AD included individuals aged 65 years or older, with an MMSE score of 26 or lower, who met the diagnostic criteria for AD based on the NINCDS-ADRDA criteria. Individuals with mild cognitive impairment, mixed dementia, vascular dementia, other neurological disorders, and head trauma were excluded from the AD group. The control group consisted of individuals aged 65 years or older, with an MMSE score greater than 27, and without a history of memory or other cognitive impairments, significant psychiatric disorders, or major coexisting medical conditions. Patients with significant comorbidities such as poorly controlled diabetes, end-stage renal failure, unstable hypertension, cancer, stroke, and end-stage cardiovascular disease were excluded from the control group. This study was approved by the Ethics Committee of the Second People’s Hospital of Yibin City (2019-069-01). Informed consent was taken from all individual participants. The RT-qPCR reactions were performed in the Gene-9660 System with SuperReal PreMix Plus. The relative quantification of mRNAs was normalized to GAPDH with the 2^−ΔΔCT^ method. The primers are listed in Table 1.

**Table 1:** The names and sequences of the PCR primers.

Primer names	Primer sequences (5’to 3’)
GAPDH-F	GGAGCGAGATCCCTCCAAAAT
GAPDH-R	GGCTGTTGTCATACTTCTCATGG
DLD-F	CTCATGGCCTACAGGGACTTT
DLD-R	GCATGTTCCACCAAGTGTTTCAT
LIAS-F	AGGAAGCTCGATGTCCCAAT
LIAS-R	TTGTAGGGCTCACTGGCATC
MTF1-F	CAGTGCGGAGAACACTTGC
MTF1-R	TGCACATAACCCTGGGACATT
PDHA1-F	CCAGTTCTGAGGCAGTGTCC
PDHA1-R	CTATGCAGGAGGCTGAGGTG

## Results

### AD-related differentially CRGs

**Figure 1:**
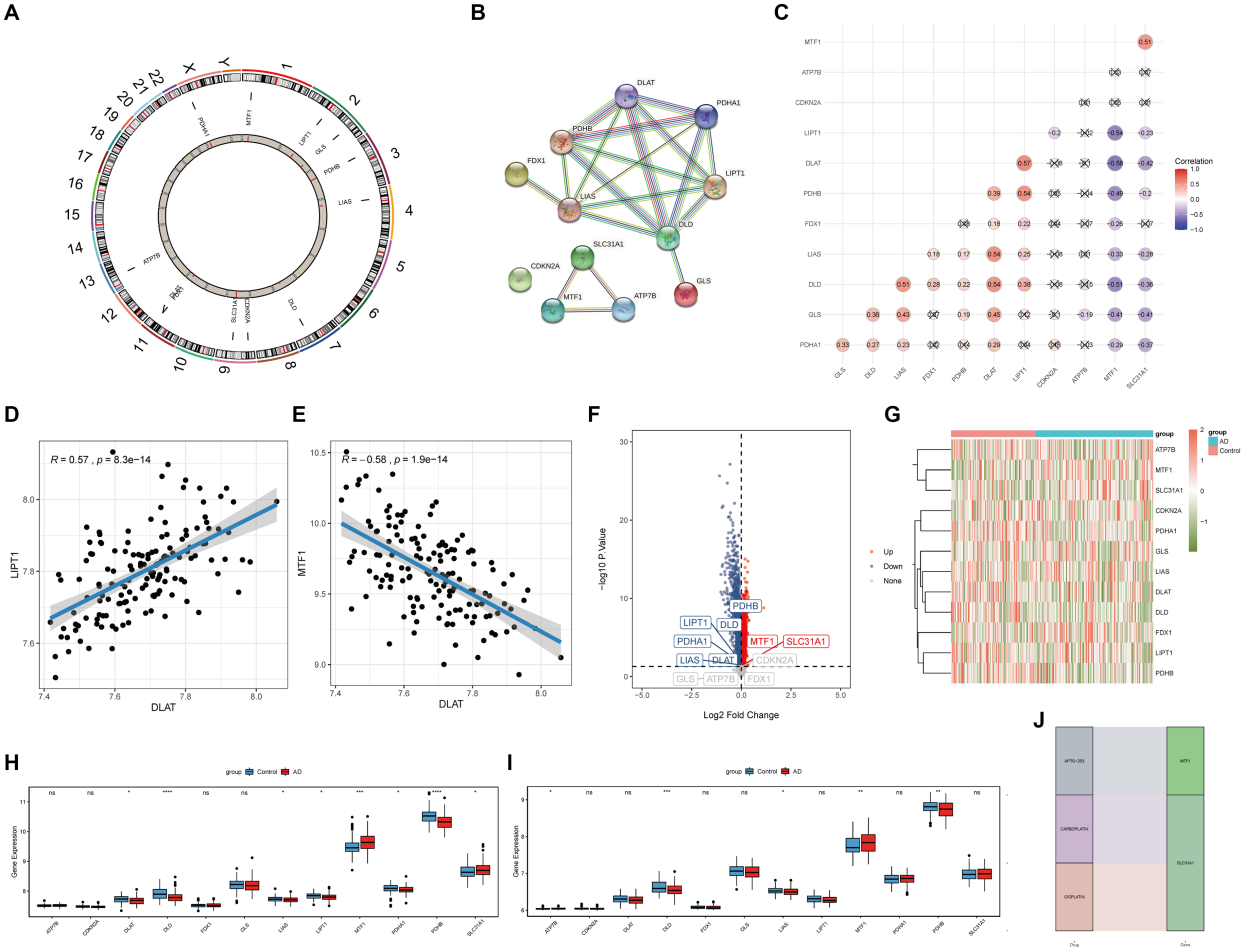
Difference in CRGs between AD and healthy samples. **(A)** The distribution of the 12 CRGs on the chromosome. **(B)** PPI network. **(C–E)** Correlation analysis between 12 CRGs. **(F)** Volcano plot. **(G)** Heat map. **(H)** Expressions of 12 CRGs in the training set. **(I)** Expressions of 12 CRGs in the validation set. **(J)** CRGs related drugs were screened from the DGIdb database. ^*^p < 0.05, ^**^p < 0.01, ^***^p < 0.001, ^****^p < 0.0001 indicated vs. Control group. CRGs, cuproptosis-related genes; AD, Alzheimer’s disease; PPI, Protein–protein interaction; DGIdb, Drug-Gene Interaction database.

The distribution of the 12 CRGs on the chromosome is shown in Figure 1A. The PPI network revealing the interactions is presented in Figure 1B. Correlation analysis results showed that LIPT1 had the highest positive correlation with DLAT (*r* = 0.57), and MTF1 had the highest negative correlation with DLAT (*r* = −0.58) (Figures 1C–E). Volcano and heat maps of CRGs between patients with AD and controls are shown in Figures 1F,G. The expression of 12 genes in the training and validation sets is shown in Figures 1H,I. Besides, CRG-related drugs were screened from the DGIdb database (Figure 1J).

### Construction of a diagnostic model

**Figure 2:**
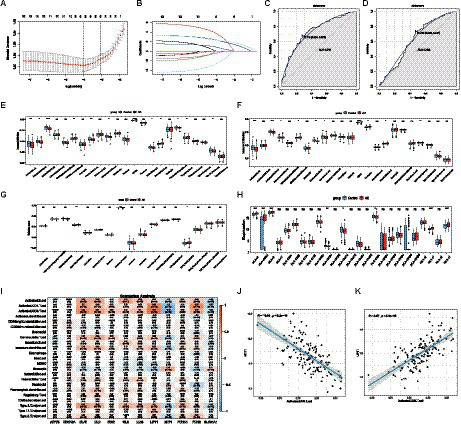
The diagnostic model and immune infiltration analysis of AD. **(A,B)** LASSO regression model was used for data dimension reduction, feature selection, and gene screening. In all, 5 CRGs, namely, CDKN2A, DLD, FDX1, PDHA1, and PDHB were screened from these 12 CRGs. **(C)** ROC curve of the AD diagnostic model in the training set. **(D)** ROC curve of the AD diagnostic model in the validation set. **(E)** The difference of 23 immune cells in the training set between the AD and healthy groups. **(F)** The difference of 23 immune cells in the validation set between the AD and healthy groups. **(G)** The difference in immune activity between the AD and healthy groups in the training set. **(H)** HLA gene differences between the AD and healthy groups in the training set. **(I)** Correlation between 23 immune cells and cuproptosis. **(J)** Relationship between activated CD8 T cells and MTF1. **(K)** Relationship between activated CD8 T cells and LIPT1. LASSO, Least Absolute Shrinkage and Selection Operator; CRGs, cuproptosis-related genes; AD, Alzheimer’s disease; ROC, receiver operator characteristic curve. HLA, Human leukocyte antigen.

With LASSO regression analysis, five genes, namely, CDKN2A, DLD, FDX1, PDHA1, and PDHB, were obtained from 12 CRGs to construct a diagnostic model (Figures 2A,B). The risk score calculating formula was as follows: RiskScore = (CDKN2A × 0.43146946) + (DLD × 1.47198071) + (FDX1 × -0.1765289) + (PDHA1 × 0.04818165) + (PDHB×1.80831198). The ROC analysis revealed that the AUC of the diagnostic model was 0.739 in the training set, and the AUC was 0.622 in the validation set, proving that cuproptosis does indeed play an important role in AD (Figures 2C,D).

### Immune infiltration characteristics in AD

The results of the ssGSEA analysis indicated that compared with the control group, the abundance of activated B cell/CD4 T cell/CD8 T cell, gamma delta T cell, immature dendritic cell, natural killer cell, plasmacytoid dendritic cell, and type 17 T helper cell were significantly reduced in the AD group, while CD56dim natural killer cells, MDSC, natural killer T cells, and regulatory T cells were significantly increased in the AD samples (Figure 2E). Consistent with the training dataset, the AD group had a lower level of infiltrated activated B cells, activated CD8 T cells, and Gamma delta T cells, whereas a higher level of infiltrated CD56dim natural killer cells, MDSC, and natural killer T cells were enriched in the validation set (Figure 2F). However, only the immunocompetence score of chemokine receptors, interferon receptors, and TGFb family members were significantly different, indicating that the change in the immune microenvironment may be one of the causes of AD (Figure 2G). A similar trend was observed for HLA gene expression (Figure 2H). The results showed that the expression levels of HLA-A, HLA-A29.1, HLA-B, HLA-F, and HLA-H in the AD group were significantly higher than those in the control group, while the expression levels of HLA-DPB1, HLA-DQA1, and HLA-DRA were significantly lower than those in the control group. Subsequently, correlation analysis between CRGs and immune cells showed that MTF1 and activated CD8 T cells exhibited a strong negative correlation, while LIPT1和activated CD8 T cells presented a strong positive correlation, indicating that the decrease of activated CD8 T cells in the AD group may be closely related to the expression of MTF1 and LIPT1, which suggests that CRGs may be a remarkable factor in the regulation of molecular and immune infiltration in patients with AD (Figures 2I–K).

### Identification of two different subtypes in the AD group

**Figure 3:**
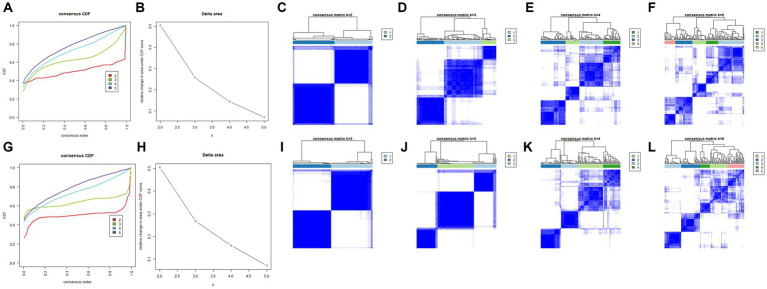
Identification of different cuproptosis modes in the AD group in the training and validation sets. **(A,B)** Consistency clustering results in the training set. **(C–F)** The consensus matrix heat map in the training set. **(G,H)** Consistent clustering results in the validation set. **(I–L)** The consensus matrix heat map results in the validation set. AD, Alzheimer’s disease.

Consensus clustering revealed that the *k* = 2 was identified with optimal clustering stability (Figures 3A,B). Then, 145 patients in the training set were clustered into two subtypes, namely, cluster A (*n* = 69) and cluster B (*n* = 76) (Figures 3C–F). Consistent with the analysis in the training set, the validation in the validation set displayed similar results (Figures 3G,H). Then, 139 patients in the validation set were clustered into two subtypes, namely, cluster A (*n* = 72) and cluster B (*n* = 67) (Figures 3I–L). Consensus clustering revealed significant differences in molecular features between the two AD subtypes.

### Diagnostic models for different AD subtypes

**Figure 4:**
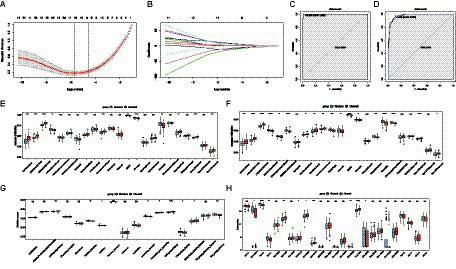
The diagnostic model and immune infiltration analysis of AD subtypes. **(A,B)** LOSSA screening results. **(C)** ROC curve of the model in the training set. **(D)** ROC curve of the model in the validation set. **(E)** The difference of 23 immune cells in the training set between the AD subtypes. **(F)** The differences of 23 immune cells in the validation set between the AD subtypes. **(G)** The difference in immune activity in the training set between the AD subtypes. **(H)** The difference in HLA genes in the training set between the AD subtypes. LASSO, Least Absolute Shrinkage and Selection Operator; CRGs, cuproptosis-related genes; AD, Alzheimer’s disease; ROC, receiver operator characteristic curve.

With LASSO regression analysis, nine genes, namely, ATP7B, DLD, GLS, LIAS, LIPT1, MTF1, PDHA1, PDHB, and SLC31A1, were obtained from 12 CRGs to construct a diagnostic model (Figures 4A,B). The risk score calculating formula was as follows: RiskScore2 = (ATP7B * -8.5997052) + (DLD * 0.9287686) + (GLS * 6.0858871) + (LIAS * 6.695944) + (LIPT1 * 1.4651665) + (MTF1 * -10.9825807) + (PDHA1 * 5.9849369) + (PDHB * 3.7707367) + (SLC31A1 * -6.1211833). The ROC analysis revealed that the AUC of the diagnostic model was 0.999 in the training set, and the AUC was 0.979 in the validation set, proving that cuproptosis does indeed play an important role in AD subtypes (Figures 4C,D).

### Immune infiltration characteristics and hypoxia states of two AD subtypes

**Figure 5:**
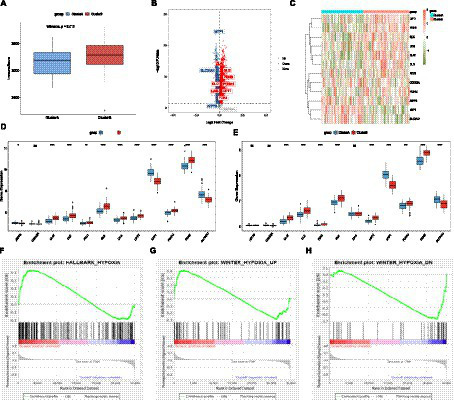
Differences in immune scores between the AD subtypes. **(A)** Differences in immune scores between the AD subtypes. **(B)** Volcano plot of differentially expressed genes (*p*-value <0.05). **(C)** Heat map. **(D)** The differences in copper death genes between the two subtypes of the experimental group. **(E)** Differences of cuproptosis genes between the AD subtypes in the validation set. **(F–H)** GSEA analysis to evaluate the hypoxia status among AD subtypes. AD, Alzheimer’s disease.

In order to explore the different immune characteristics of two AD subtypes, we observed different CRG expression landscapes through ssGSEA analysis. The results of ssGSEA analysis indicated that compared with Cluster A, activated B cell/CD4 T cell/CD8 T cell, gamma delta T cell, immature dendritic cell, regulatory T cell, type 1 T helper cell, and type 2 T helper cell in Cluster B were significantly increased, while activated dendritic cell, MDSC, monocyte, and neutrophil were remarkably decreased. Consistent with the training dataset, the alternations of activated B cell/CD4 T cell/CD8 T cell/dendritic cell, gamma delta T cell, immature dendritic cell, MDSC, monocyte, neutrophil, type 1 T helper cell, and type 2 T helper cell were identical in the validation set (Figures 4E,F). Cytokine receptors, cytokines, interferon receptors, interleukin receptors, TGFb family members, and TNF family members were more active in Cluster A, while BCR signaling pathway, natural killer cell cytotoxicity, TCR signaling pathway, and TGFb family member receptors were more active in Cluster B (Figure 4G). Because of HLA-related genes, Cluster B exhibited lower proportions of HLA-A29.1, HLA-B, HLA-C, HLA-DQB2, and HLA-DRB1, whereas the expression of HLA-DMB, HLA-DOB, HLA-DPA1, HLA-DPB1, and HLA-DRA was relatively increased in Cluster B (Figure 4H). The immunescore of each patient with AD, calculated according to the ESTIMATE algorithm, showed that a significantly higher immunoscore was detected in cluster B, suggesting that there are different immune microenvironments between the two subtypes of AD, and B may have a higher level of immune infiltration (Figure 5A). Heat map and volcano map of DEGs between the two AD subtypes are shown in Figures 5B,C. To explore the relationship between two AD subtypes and cuproptosis, the expression levels of 12 CRGs were compared between cluster A and cluster B (Figure 5D). ATP7B, SLC31A1, and one anti-cuproptosis gene, namely, MTF1, were significantly low expressed, while GLS and seven pro-cuproptosis genes, namely, DLAT, DLD, FDX1, LIAS, LIPT1, PDHA1, and PDHB, were significantly high expressed in the cluster B (Figure 5E). The results showed that compared with group A, the expression of CRGs in cluster B changed more significantly and the level of immune infiltration was higher, suggesting that the different immune microenvironment between the two AD subtypes may be caused by the different expression levels of CRGs.

GSEA analysis indicated that the gene sets HALLMARK_HYPOXIA and WINTER_HYPOXIA_UP were upregulated in cluster A, while gene set WINTER_HYPOXIA_DN was significantly upregulated in cluster B, indicating that the degree of hypoxia in the cluster A of the AD group was higher (Figures 5F–H).

### WGCNA and identification of hub genes

**Figure 6:**
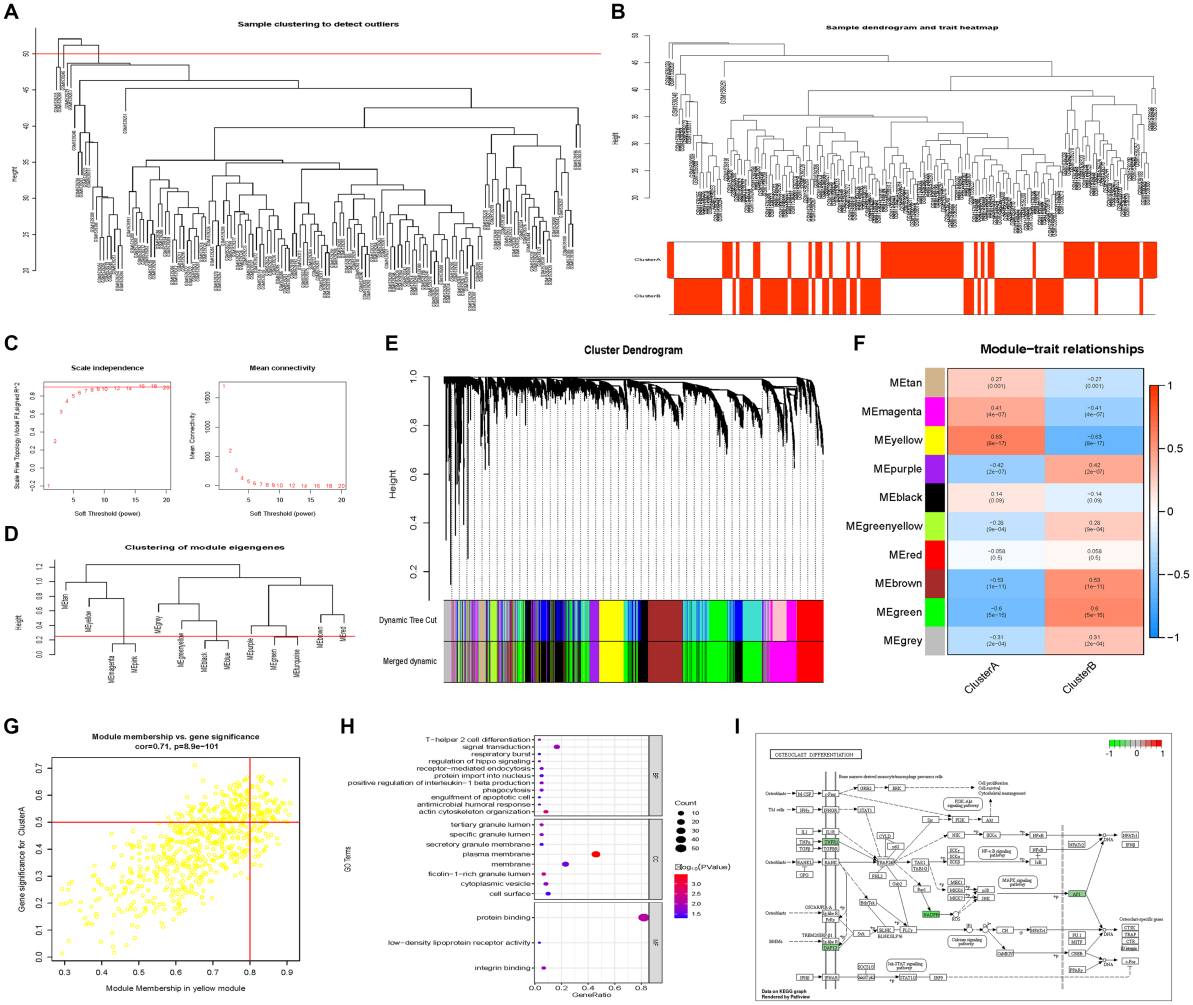
Co-expression network constructions by WGCNA and enrichment results. **(A)** Sample screening dendrogram. **(B)** Sample dendrogram and feature heat map. **(C)** The scale-free fitting index of different soft threshold powers (β) and the average connectivity of various soft threshold powers. **(D)** Module merge. **(E)** Genes are divided into different modules by hierarchical clustering, and different colors represent different modules. **(F)** Heat map showing the correlation between module characteristic genes and the two AD models. **(G)** Scatter plot. **(H)** GO enrichment results. **(I)** KEGG enrichment results. AD, Alzheimer’s disease; WGCNA, Weighted gene co-expression network analysis; GO, Gene Ontology; KEGG, Kyoto Encyclopedia of Genes and Genomes.

142 samples were screened out through strict quality control procedures (Figures 6A–B). The power *β* = 7 was selected as the soft threshold to build a scale-free network (Figure 6C). A total of 10 modules were determined, namely, the tan module, magenta module, yellow module, purple module, black module, green-yellow module, red module, brown module, green module, and grey module (Figures 6D,E). The correlation between module eigengenes and AD subtypes was analyzed, indicating that the yellow module, including 650 genes, was the most model associated with the two subtypes, with a correlation coefficient of 0.63 (Figure 6F). With |GS| > 0.5 and |MM| > 0.8, 71 genes were identified as AD subtype-associated genes (Figure 6G). Then, 71 intersection genes were obtained by overlapping AD subtype-associated genes and DEGs between two subtypes. These genes were then uploaded to DAVID for GO/KEGG analyses, and the significance level was set at a *p*-value <0.05. GO biological process (BP), GO cellular component (CC), GO molecular function (MF), and KEGG pathways were significantly enriched (Figures 6H,I). In CC terms, the DEGs were mainly involved in the plasma membrane, membrane, and cell surface. In MF terms, the DEGs were mainly associated with protein binding, integrin binding, and low-density lipoprotein receptor activity. In BP terms, the DEGs were mainly enriched in signal transduction, actin cytoskeleton organization, and positive regulation of interleukin-1 beta production, phagocytosis, and receptor-mediated endocytosis (Figure 6H). KEGG pathway analysis indicated that the DEGs were mainly related to osteoclast differentiation (Figure 6I).

### Drug prediction and molecular docking

**Figure 7:**
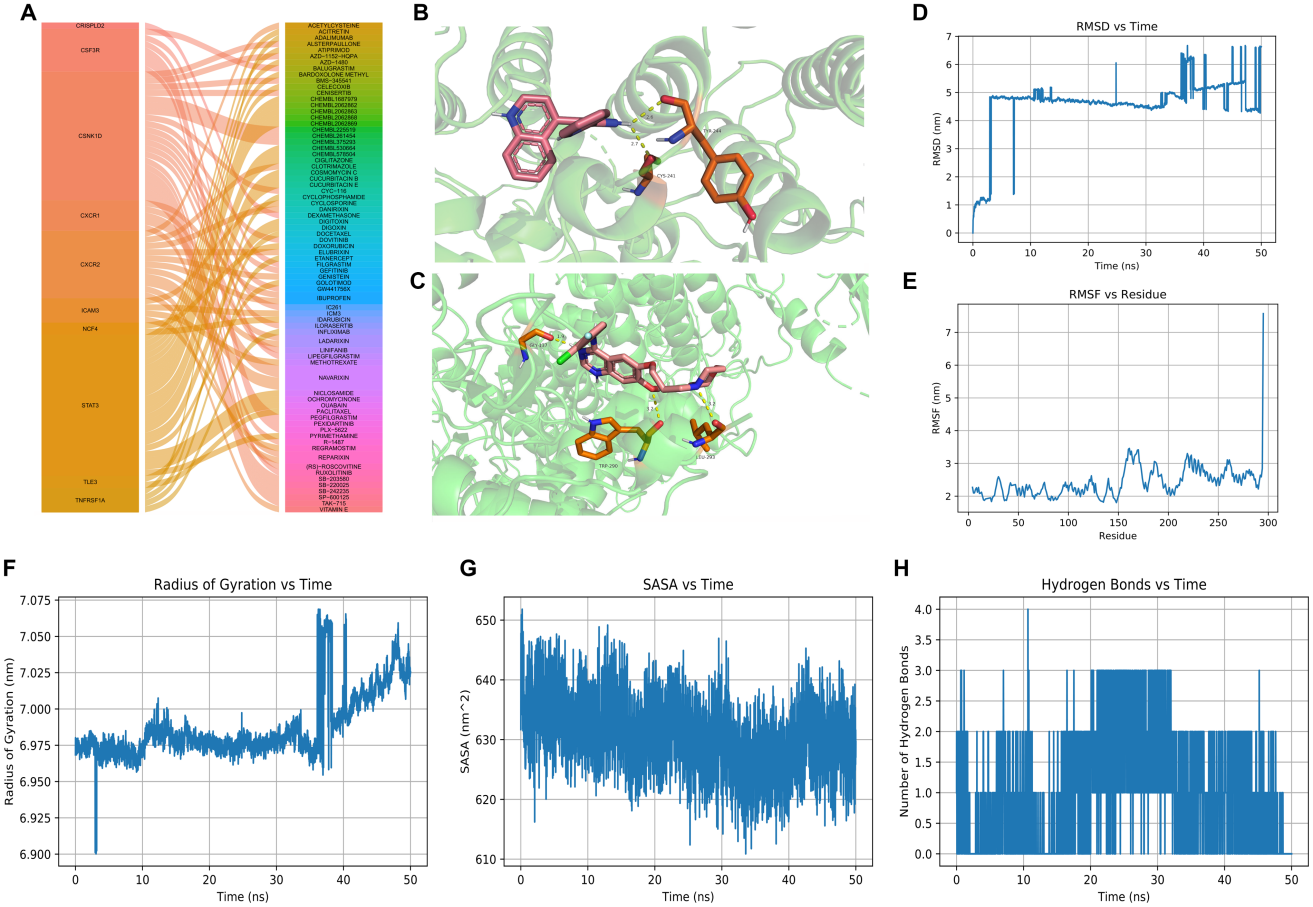
Drug predictions, molecular docking, and molecular dynamics simulation. **(A)** Drug predictions based on the DGIdb database. **(B)** Molecular docking to verify that CHEMBL261454 had a significant role in the regulation of CSNK1D. **(C)** Molecular docking to verify that GEFITINIB had a lower role in the regulation of CSNK1D. **(D–H)** Molecular dynamics simulation results including RMSD, RMSF, radius of gyration, SASA, and hydrogen bonds of CSNK1D and CHEMBL261454. RMSD, root mean square deviation; RMSF, root mean square fluctuation; SASA, solvent accessible surface area.

By overlapping 71 intersection genes and DEGs between AD and control groups, 60 genes were obtained to predict the potential drugs for AD, and finally, 10 genes (CSNK1D, CRISPLD2, CSF3R, CXCR1, CXCR2, ICAM3, NCF4, STAT3, TLE3, and TNFRSF1A) were observed as targets of the 74 predicted drugs (Figure 7A). Among them, CSNK1D was confirmed to be highly expressed in AD and its inhibitor, CHEMBL261454, has been shown to have therapeutic effects on AD. Furthermore, CHEMBL261454 was selected for further molecular docking analysis with CSNK1D to verify if CHEMBL261454 and its metabolites had a significant role in the regulation of CSNK1D. The results showed that CHEMBL261454 had strong interactions with CSNK1D (−4.47 kcal/mol) (Figure 7B). Moreover, the molecular docking analysis between CSNK1D and another drug, GEFITINIB, was conducted, exhibiting a binding energy of −3.24 kcal/mol (Figure 7C), which was higher than that between CSNK1D and CHEMBL261454. It was further demonstrated that CSNK1D and CHEMBL261454 may play a crucial role in the development of AD.

### MD simulation

MD simulation has been widely used to evaluate the structural characteristics of the protein-ligand systems and study the binding stability between the proteins and the molecules. In the present study, MD simulation was performed to simulate the binding stability of CSNK1D and CHEMBL261454. Analysis of molecular dynamics simulation results can be seen in Figures 7D–H.

The RMSD curve depicted the fluctuation of molecular conformation after the docking of small molecule-protein complexes. It tended to stabilize within 10–30 ns, with slight fluctuations observed after 30 ns, indicating a relatively stable binding (Figure 7D). The RMSF curve reflected the fluctuation of residues within the molecule, where RMSF values suggest potential instability of the protein-ligand complex (Figure 7E). The RoG curve tended to reach equilibrium, indicating a stable conformation (Figure 7F). The SASA can be used to describe the effective interaction between ligand complexes and receptors (Figure 7G). Compounds with high SASA values tended to form unstable protein-ligand complexes due to their easy access to solvent, while complexes with low SASA values were considered stable. Overall, there was a decreasing trend, indicating that protein-ligand complexes may become more stable over time. H-bonds facilitated the binding ability between proteins and ligands, and the number of hydrogen bonds could reflect the induced binding affinity. On average, protein-ligand complexes formed two hydrogen bonds (Figure 7H). In brief, these results showed that CSNK1D could be well combined with CHEMBL261454.

### Verification of gene expression by RT-qPCR

**Figure 8:**
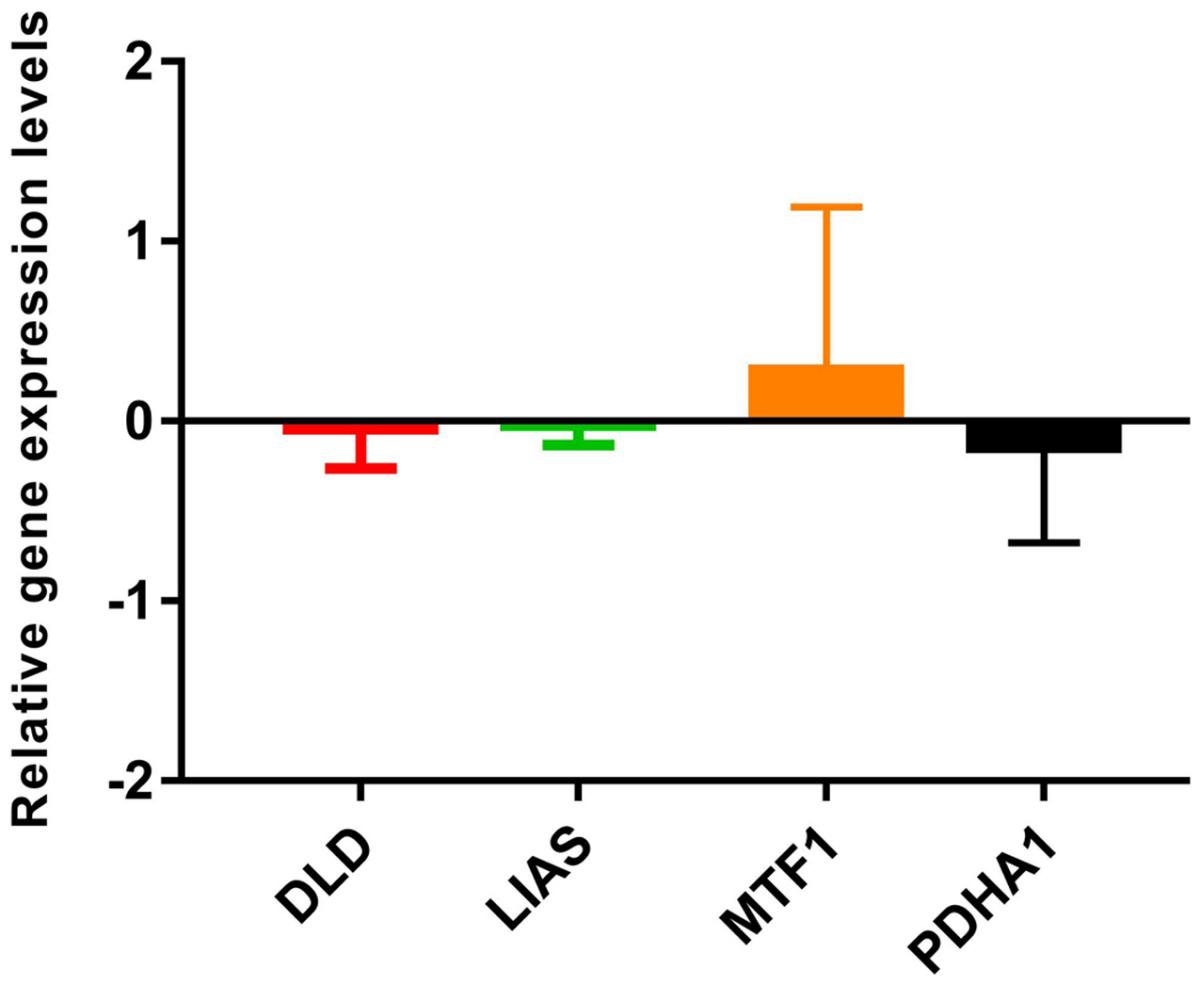
Verification of four selected gene expressions by RT-qPCR.

In total, four genes (DLD, LIAS, PDHA1, and MTF1) were selected for RT-qPCR analysis. Based on our integrated analysis, DLD, LIAS, and PDHA1 were downregulated while MTF1 was upregulated. It was noted that the RT-qPCR results were in line with our integrated analysis (Figure 8).

## Discussion

Toxic oligomeric pTau, amyloid beta peptide, and neurofibrillary tangles and plaques accumulated in the brain are diagnostic features of advanced AD (Jahangir et al., 2014; Ahmad et al., 2017). As is well-known, pyruvate dehydrogenase complex (PDHc) and α-ketoglutarate dehydrogenase complex (αKGDHc) are indispensable enzymes in the Krebs cycle of glucose metabolism (Sang et al., 2018). The pyruvate dehydrogenase is produced from the PDHA1 gene and is involved in the energy metabolic process. A recent study proposed for the first time that the PDHA1 expression was most significantly changed in AD mice compared to controls (Yang et al., 2020). Similarly, the relationship between PDHA1 and Parkinson’s disease (PD) has been revealed (Miki et al., 2017). The DLD gene encodes the E3 subunit (dihydrolipoamide dehydrogenase) of the α-KGDHc enzyme, which is composed of three subunits. In a previous study (Brown et al., 2004), a positive correlation between the DLD gene and AD was proved. Inherited mutations in the DLD gene are also associated with metabolic diseases that often present with severe neurological symptoms (Hong et al., 1997). Currently, 14 disease-causing substitutions in the DLD gene have been documented in the literature (Ambrus, 2019). A genome scan research of families with late-onset AD (LOAD) (Brown et al., 2004) identified a marker that is located within 5 cm of the DLD locus (Pericak-Vance et al., 2000). In this study, PDHA1 and DLD expression levels were downregulated in patients with AD, and RT-qPCR results showed the same trend, which the abovementioned evidence suggests may provide new insights into the treatment of AD.

The amyloid b peptide (Ab) is generally considered the primary culprit in AD (Selkoe, 2001) and is the main component of amyloid plaques. Amyloid plaques are known to be highly enriched for zinc, copper, and iron ions (Danielsson et al., 2007). Chelating of aggregation-promoting or redox-active metal ions, such as zinc and copper, or reducing oxidative stress can be the methods to reduce the toxicity of Ab peptides. Copper homeostasis is disrupted in the brains of patients with AD, resulting in copper enrichment in the amyloid plaques and decreased overall levels of copper, which leads to reduced Cu/Zn SOD-1 activity (Lovell et al., 1998). In a transgenic mouse model of AD, dietary copper has been found to restore the function of SOD-1 and reduce plaque formation (Bayer et al., 2003). Metal chelators have also been shown to be effective, and the expression of metal response element (MRE)-binding transcription factor-1 (MTF-1), the key regulator of metal homeostasis, or of human metallothioneins, can reduce Ab generation. MTF-1 is the most noticeable metal-dependent transcription factor, activated by various stimuli, including copper and zinc, and binding to MREs to regulate the expression of copper detoxification metallothioneins (Liuzzi et al., 2004). Since MTF-1 plays a crucial role in defense against several cell stress conditions, including oxidative stress and hypoxia, and has been effective in Drosophila models of PD and AD (Saini et al., 2011), making it clear if MTF-1 gene expression modulates the course of human neurodegenerative disorders, including AD, helps shed more light on the molecular mechanisms of AD pathogenesis. In the present study, MTF-1 expression was upregulated in AD according to both bioinformatics analysis and biological experiments, copper homeostasis was disrupted, and copper was enriched in Ab, contributing to the onset and progression of the AD phenotype.

In order to study the relationship between hypoxia and different subtypes of AD related to copper death, we selected a set of hypoxia-related genes for GSEA analysis and found that the degree of hypoxia in group A was particularly significant, indicating that there was differentiated hypoxia in different subtypes of AD. In the central nervous system, HIF is mainly involved in the regulation of neurogenesis, neuronal differentiation, and neuronal apoptosis. It has been found that the decrease in HIF-1a level is related to the increase of tau protein phosphorylation and neurofilament formation (Merelli et al., 2018). Pharmacological activation of HIF-1 has a neuroprotective effect on AD, so it may be used for treatment (Guo et al., 2017; Merelli et al., 2018). In clinical trials of patients with AD, it is reported that HIF-1 inducer deferoxamine (DFO) slows down the decline of cognitive ability (Zhang et al., 2011). However, it must be noted that HIF-1 may also have a negative impact on AD. For example, in SK-N-MC cells, HIF-1α activates the production of Aβ through Akt–mTOR-HIF-1α and Akt-NF-κB pathways (Kim et al., 2017). Therefore, the role of the HIF signaling pathway in the development of AD-related neurodegeneration is still controversial and needs further study.

Recent research suggests that the brains of individuals with AD are marked by an immunosuppressive microenvironment (Salminen et al., 2018). This immunosuppression is a key feature of pathological disorders that involve chronic inflammation. Immunosuppressive factors, such as IL-10, TGF-β, and ROS, are produced by immunosuppressive cells, such as MDSCs, and can inhibit the functions of many immune cells, such as dendritic cells, macrophages, CD4/CD8T, and B cells (Salminen, 2021a,b). Immunosuppressive MDSCs can be recruited via chemotaxis into inflamed tissues where their proliferation and activation could be enhanced by mounting inflammatory mediators (Salminen et al., 2018). Similarly, in our study, we observed that MDSCs, CD56dim natural killer cells, and natural killer T cells were significantly elevated in patients with AD. Conversely, gamma delta T cells, activated CD8 T cells, and activated B cells were significantly reduced both in patients with AD and healthy subjects. Previous research has demonstrated that patients with AD exhibit a significant reduction in CD8+ T cell subsets, a significant increase in CD4+ helper T cells, and a significant correlation between the CD4/CD8 ratio and cognitive decline that is characteristic of AD (Unger et al., 2020). Therefore, the development and progression of AD are intimately linked to the immune infiltration of various immune cells. Identifying the intrinsic mechanism of neuroimmunity is significant for the prevention and treatment of AD. Meanwhile, the expression of the HLA gene showed the same trend. Compared with the healthy group, the expression of the HLA-I gene in the AD group was significantly increased, while the expression of the HLA-II gene was significantly decreased. The expression level of HLA-I genes is mainly related to rejection, which can induce CD8 + T cells to activate, while the expression level of HLA-II genes is mainly related to humoral immunity, which can induce CD4 + T cells to activate and stimulate B cells to produce specific antibodies. Recently, a large-scale genome-wide association meta-analysis confirmed that the haplotype HLA-dr15 mediated by nerve and immunity is a risk factor for delayed Alzheimer’s disease through the fine localization of the human leukocyte antigen (HLA) region (Kunkle et al., 2019). By examining millions of polymorphisms in subjects, studies have revealed that genes such as HLA-DRB5-DBR1 are closely related to the risk of AD (Karch and Goate, 2015; Hampel et al., 2020). Therefore, in short, the occurrence and development of AD are closely related to the immune infiltration level of various immune cells. It is of great significance to clarify the internal mechanism of neuroimmunity for the prevention and treatment of AD.

Decades of research have produced increasing advances in antineurodegenerative therapies, whereas traditional classifications based on histology show frequent resistance (Nandigam, 2008). Thus, the identification of more suitable molecular clusters benefits the individualized treatment of AD. To differentiate patients with AD from healthy subjects, we constructed a diagnostic model for AD based on the CDKN2A, DLD, FDX1, PDHA1, and PDHB genes. Because the two subtypes of AD have different levels of immune infiltration and hypoxia, in order to guide clinical medication more accurately, we also screened nine genes, namely, ATP7B, LIAS, DLD, GLS, MTF1, LIPT1, PDHA1, SLC31A1, and PDHB, in order to distinguish the two subtypes of AD, and constructed a diagnostic model based on these genes. The AUC value of RiskScore2 indicated that this model was effective in distinguishing between the two different AD subtypes. This model represents a promising diagnostic tool and provides new insights for the diagnosis of AD. Furthermore, we analyzed separately the correlation of immune infiltration in the two subtypes of AD. Our findings suggested that the proportion of γδT cells, activated CD4 T cells, activated B cells, activated dendritic cells, activated CD8 T cells, and immature dendritic cells was higher in both the experimental and validation sets than in the Cluster A group. However, the proportion of MDSCs, monocytes, neutrophils, type 1 helper T cells, and type 2 helper T cells was lower. These results suggested that the two AD subtypes may exhibit distinct neuroimmune characteristics and that timely recognition of these subtypes may facilitate the targeted treatment of AD.

The stress-induced protein kinase CK1 delta (CSNK1D) lies in the long arm of chromosome 17 (17q25.3) in humans, encoding CK1δ, a member of the CK1 family (Graves et al., 1993). Increasing studies have demonstrated that the dysregulation and activity of CK1δ expression are not only found in various cancers but also in different neurological diseases including AD (Zhu et al., 2022; Jin et al., 2023). Hence CSNK1D is expected to be a promising treatment target for AD. CHEMBL261454, as an inhibitor of CSNK1D, may have a certain effect on AD13. Furthermore, CHEMBL261454 was selected for further molecular docking analysis along with CSNK1D. Molecular docking was used to verify if CHEMBL261454 and its metabolites had a significant role in the regulation of CSNK1D. As a control, another drug, GEFITINIB, was subjected to the same procedure as CHEMBL261454. The results showed that CHEMBL261454 had strong interactions with CSNK1D (docking score − 4.47 kcal/mol), suggesting that they might play vital roles in the development of AD, which was further validated by MD simulation.

Some limitations of this study need to be noted. First, the performance of the diagnostic model needs to be confirmed by more detailed and accurate clinical materials. Second, more AD samples will be collected to prove the accuracy of AD clusters as well as the potential correlation between CRGs and immune responses. Additionally, molecular docking and MD simulation can be valuable tools in the validation process for drug predictions, but further related experiments will be needed to provide additional insights and predictions.

## Conclusion

In summary, two distinct patterns of cuproptosis were identified in patients with AD, which were divided into two subtypes, A and B. Based on the overall analysis of patients with AD, the immune infiltration and hypoxia stress of the two subtypes of AD were analyzed, disclosing the correlation between CRGs and immune cells and elucidating the significant heterogeneity of immune between patients of the two AD clusters. Moreover, the diagnostic model of AD subtypes was established. Currently, only limited drugs targeting these key genes are expected to alleviate AD, suggesting that more convincing research needs to be performed.

## Data availability statement

The original contributions presented in the study are included in the article/Supplementary material, further inquiries can be directed to the corresponding authors.

## Ethics statement

The studies involving humans were approved by the Ethics Committee of the Second People’s Hospital of Yibin City (2019-069-01). The studies were conducted in accordance with the local legislation and institutional requirements. The participants provided their written informed consent to participate in this study.

## Author contributions

BN, LJ, and YD: conception and design of the study. LJ, YD, XZ, and BN: provision of study materials. XX, SS, ZF, BN, and YD: collection and assembly of data. LQ, BN, and SS: data analysis and interpretation. All authors: manuscript writing and final approval of manuscript.

## Footnotes

^1^ www.ncbi.nlm.nih.gov/geo

^2^ https://string-db.org/

^3^ http://www.immport.org

^4^ https://dgidb.org/

^5^ http://www.rcsb.org/pdb/home/home.do
